# Symptomatic Osteoarthritis May Be Associated with Higher Symptom Burden and Thrombotic Risk in Patients with Myeloproliferative Neoplasms: A Prospective Two-Centre Study

**DOI:** 10.3390/jcm15145663

**Published:** 2026-07-19

**Authors:** Hrvoje Holik, Ivan Krecak, Marko Lucijanic, Ivan Samardzic, Danijel Pilipac, Ivana Vucinic Ljubicic, Bozena Coha, Alma Kitter Pipic, Blazenka Miskic, Silva Zupancic-Salek

**Affiliations:** 1 Department of Internal Medicine, ‘Dr. Josip Bencevic’ General Hospital, 35000 Slavonski Brod, Croatia; 2 Faculty of Medicine, Josip Juraj Strossmayer University of Osijek, 31000 Osijek, Croatia; 3 Department of Internal Medicine, General Hospital of Sibenik-Knin County, 22000 Sibenik, Croatia; 4 Faculty of Medicine, University of Rijeka, 51000 Rijeka, Croatia; 5 University of Applied Sciences, 22000 Sibenik, Croatia; 6 University Hospital Dubrava, 10000 Zagreb, Croatia; 7 Department of Scientific Research and Translational Medicine, Clinical Hospital Dubrava, 10000 Zagreb, Croatia; 8 Faculty of Medicine, University of Zagreb, 10000 Zagreb, Croatia; 9 Department of Orthopedic Surgery, ‘Dr. Josip Bencevic’ General Hospital, 35000 Slavonski Brod, Croatia; 10 Special Hospital for Orthopedics, 23210 Biograd na Moru, Croatia; 11 Department of Laboratory Diagnostics, ‘Dr. Josip Bencevic’ General Hospital, 35000 Slavonski Brod, Croatia; 12 Faculty of Dental Medicine and Health Osijek, Josip Juraj Strossmayer University of Osijek, 31000 Osijek, Croatia; 13 Department of Hematology and Coagulation, University Hospital Holy Spirit, 10000 Zagreb, Croatia

**Keywords:** osteoarthritis, myeloproliferative neoplasms, symptom burden, thrombosis

## Abstract

**Background/Objectives:** *BCR::ABL1*-negative myeloproliferative neoplasms (MPNs), including essential thrombocythemia (ET), polycythemia vera (PV), and myelofibrosis (MF), are clonal hematopoietic stem cell disorders characterized by chronic systemic inflammation and elevated thrombotic risk. Osteoarthritis (OA), the most prevalent joint disease globally, is common in MPNs and shares a common proinflammatory cytokine milieu with MPNs. However, whether symptomatic OA may independently impact MPN-related symptom burden and thrombotic outcomes remains unexplored. **Methods:** In this prospective two-center study conducted in Croatia (2021–2023), 107 consecutive MPN patients diagnosed by 2016 World Health Organization criteria underwent orthopedic evaluation and completed the MPN Symptom Assessment Form (MPN-SAF) at enrolment. Symptomatic OA was defined as radiographic grade ≥1 (Kellgren–Lawrence) with concordant symptoms. Patients were subsequently followed for thrombotic events. Multiple linear regression and Cox proportional hazards regression were used for multivariable analyses. **Results:** Symptomatic OA was identified in 64 patients (59.8%). The total symptom score (TSS) was significantly higher in OA patients (*p* < 0.001), and in multivariable analysis, OA independently predicted higher TSS (β 10.12, 95% confidence interval-CI 5.38–14.86, *p* < 0.001), alongside female sex and arterial hypertension. Over a median follow-up of 44 months, 10 thrombotic events occurred. Patients with symptomatic OA had significantly worse time to thrombosis (hazard ratio-HR 3.61, 95% CI 1.02–12.8, *p* = 0.046). In multivariable Cox regression, OA remained associated with thrombosis (HR 15.55, *p* = 0.002), while female sex (HR 0.17, *p* = 0.042) and aspirin use (HR 0.15, *p* = 0.019) were protective. **Conclusions:** Symptomatic OA may be associated with higher symptom burden and, in this preliminary analysis, with higher thrombotic risk in MPNs. These findings support systematic OA evaluation in MPNs and multidisciplinary management strategies targeting this frequent and burdensome comorbidity.

## 1. Introduction

*BCR::ABL1*-negative myeloproliferative neoplasms (MPNs)—comprising essential thrombocythemia (ET), polycythemia vera (PV), and myelofibrosis (MF)—are clonal hematopoietic stem cell disorders characterized by excessive myeloproliferation, chronic inflammation, and markedly elevated cardiovascular risk [1]. These conditions are driven by somatic mutations in Janus Kinase 2 (*JAK2*), calreticulin (*CALR*), or thrombopoietin gene receptor (*MPL*) genes, which cause constitutive activation of the JAK-STAT signaling pathway, resulting in sustained elevation of proinflammatory cytokines and a prothrombotic milieu that underlies the development of arterial and venous thrombosis [1,2]. Beyond thrombohemorrhagic events, MPN patients also carry a substantial symptom burden that profoundly impairs quality of life, systematically captured by the MPN Symptom Assessment Form (MPN-SAF) [3,4].

Osteoarthritis (OA), the most prevalent form of arthritis globally, is a progressive degenerative joint disease driven by ageing, obesity, mechanical loading, and chronic low-grade inflammation [5,6]. The proinflammatory cytokines centrally implicated in OA-related cartilage degradation and synovial inflammation—including interleukin (IL)-1β, IL-6, IL-15, and tumor necrosis factor (TNF)-α—are the same mediators that characterize the inflammatory milieu of MPNs [5,6,7]. Given that MPNs predominantly affect older adults and are associated with systemic inflammation and a broad cardiometabolic risk profile, MPN patients may be disproportionately susceptible to OA development [1,8]. Among the chronic inflammatory conditions that could interact with MPN biology, OA was specifically chosen because it is the single most prevalent joint disease worldwide, shares an overlapping proinflammatory cytokine profile with MPNs, is readily assessable through standardized clinical and radiographic criteria, and, unlike many other chronic inflammatory disorders, is amenable to targeted interventions (i.e., weight reduction, physical rehabilitation, joint-directed therapy), making it a particularly relevant and actionable comorbidity to investigate in this patient population. This hypothesis was formally investigated in our prior cross-sectional study [8], which demonstrated a high prevalence of symptomatic and radiologically confirmed hip and/or knee OA among MPN patients (61%), compared to the general population. In that study, advanced age and higher body weight were identified as independent risk factors for symptomatic OA, while cytoreductive treatment exerted a protective effect. Furthermore, OA patients more frequently harbored the *JAK2* mutation, had worse performance status, and a greater history of prior thrombosis, suggesting that OA may present more frequently within a more aggressive MPN disease biology.

On the other hand, whether OA independently may worsen MPN-related outcomes—rather than simply co-occurring with a more severe disease phenotype—remains unexplored. In the present study, conducted in the same but extended patient cohort, we investigated how symptomatic OA may impact specific MPN symptoms and whether it may confer an increased risk of thrombosis during follow-up.

## 2. Patients and Methods

### 2.1. Study Design and Patient Selection

This was a prospective two-center study conducted at two community hospitals in Croatia (Dr. Josip Bencevic General Hospital Slavonski Brod, and the General Hospital of Sibenik-Knin County) between 2021 and 2023. Consecutive MPN patients diagnosed according to the 2016 World Health Organization (WHO) criteria [2] were enrolled. Patients younger than 18 years, pregnant women, those with prior joint trauma or infection, and patients with other active malignancies were excluded from participation. The study was approved by the Ethics Committees from both participating centers, and all participants provided written informed consent in accordance with the Declaration of Helsinki (detailed Ethics Committees approval dates and numbers are listed at the end of the article).

### 2.2. Osteoarthritis Evaluation

At study entry, all participants underwent clinical evaluation by an orthopedic surgeon. Patients with suspected symptomatic hip and/or knee OA (joint pain, swelling, crepitus, or restricted range of motion) underwent plain radiography of the affected joint(s). Orthopedic surgeons were blinded for MPN characteristics. OA was defined as radiographic grade ≥1 according to the Kellgren–Lawrence classification [9] in the presence of concordant symptoms. OA status was assessed only once, at study entry; patients were not systematically re-evaluated for new-onset or progressive OA during follow-up.

### 2.3. Symptom Assessment

All participants completed the MPN-SAF [3,4] at study entry. The MPN-SAF is a validated, internationally standardized patient-reported outcomes tool comprising 10 items (fatigue, early satiety, abdominal discomfort, inactivity, concentration problems, night sweats, itching, bone pain, fever, and unintentional weight loss) scored on a 0–10 numerical scale, generating a Total Symptom Score (TSS) ranging from 0 to 100.

### 2.4. Statistical Analyses

After initial diagnostic workup for OA, all MPN patients were observed for the occurrence of thrombotic events. Time to thrombosis (TTT) was measured from the date of OA diagnosis until the first arterial (myocardial infarction, transient ischemic attack, stroke, or peripheral arterial occlusion) or venous thrombotic event (deep vein thrombosis and/or pulmonary embolism). Death was treated as a censoring event. The Shapiro–Wilk test was used to assess data distribution. Differences in categorical variables between groups were analyzed using the Fisher’s exact or the chi-square test, whereas continuous variables were compared with the student’s *t*-test or the Mann–Whitney U test, depending on data distribution. Multiple linear regression was used to assess the independent association of symptomatic OA with TSS. Survival analyses were performed using the Kaplan–Meier method, the log-rank test, and Cox proportional hazards regression. Statistical analyses were performed using licensed MedCalc Statistical Software^®^ (version 20.216, Ostend, Belgium). Additional sensitivity analyses were performed in Python (version 3.12, Python Software Foundation, Beaverton, OR, USA), using the lifelines (v0.30.3) and scikit-survival (v0.28.0) packages (all open-source scientific Python libraries). These included: (i) ridge (L2)-penalized Cox regression, with coefficient shrinkage evaluated across a range of pre-specified penalty strengths; (ii) elastic-net penalized Cox regression (L1/L2 mixing parameter = 0.5), with the regularization strength selected by 5-fold cross-validated Harrell’s concordance index, allowing simultaneous shrinkage and variable selection; (iii) bootstrap resampling (1000 iterations, stratified by event status to preserve the original number of thrombotic events in each resample) of the original, non-penalized multivariable Cox model, generating empirical percentile-based 95% confidence intervals for the hazard ratio of OA; and (iv) a pre-specified parsimonious multivariable model restricted to OA, age, sex, and aspirin use, corresponding to a more favorable events-per-variable ratio (≈3.3). Model discrimination for each multivariable Cox model was quantified using Harrell’s concordance index (C-index), both as apparent (in-sample) values and after bootstrap optimism correction (1000 resamples); calibration was assessed using a bootstrap-derived calibration slope, obtained by refitting a Cox model with the bootstrap-derived linear predictor as the sole covariate in the original dataset. The proportional hazards assumption for each multivariable Cox model was formally tested for each covariate using Schoenfeld residuals (rank-transformed time). A two-sided *p*-value < 0.05 was considered statistically significant for all presented analyses.

## 3. Results

### 3.1. Patient Characteristics

A total of 107 MPN patients were included (ET, *n* = 44, PV, *n* = 35, MF, *n* = 28); the median age was 68 years (range 35–90), 54.2% were female, and symptomatic OA of any localization was identified in 64 patients (59.8%). As detailed in Table 1, symptomatic OA was significantly associated with older age (*p* < 0.001), presence of the *JAK2* mutation (*p* = 0.018), prior thrombosis (*p* = 0.018), higher body weight (*p* < 0.001), higher body mass index (BMI; *p* < 0.001), higher serum uric acid (*p* = 0.001), lower estimated glomerular filtration rate (eGFR; *p* = 0.007), lower hemoglobin (*p* = 0.004) and hematocrit levels (*p* = 0.029), arterial hypertension (*p* = 0.008), hyperlipidemia (*p* = 0.008), and greater use of nonsteroidal anti-inflammatory drugs (NSAIDs; *p* = 0.015), opioids (*p* = 0.026), statins (*p* = 0.005), and angiotensin-converting enzyme (ACE) inhibitors (*p* = 0.006).

### 3.2. Impact of Osteoarthritis on MPN Symptom Burden

**Figure 1:**
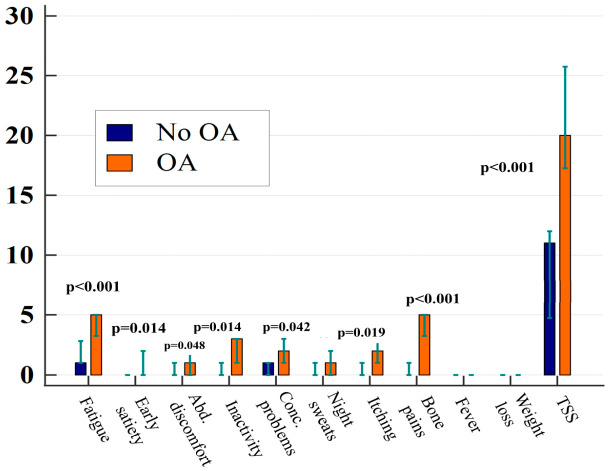
Associations of any OA with disease-specific symptoms in patients with chronic myeloproliferative neoplasms (MPNs) assessed with the MPN-SAF (Myeloproliferative Neoplasm Symptom Assessment Form).

The median TSS was 16 (range 0–50) and was significantly higher in patients with symptomatic OA of any localization when compared to those without OA (median score 20 vs. 11; *p* < 0.001). The majority of individual MPN-SAF items were also significantly elevated in OA patients, as shown in Figure 1; the results were consistent for TSS and all individual patient symptoms for both hip and knee OA localizations (*p* < 0.050 for all analyses). In the multivariable linear regression analysis, the presence of symptomatic OA of any localization (β 10.12, 95% CI 5.38–14.86, *p* < 0.001), female sex (β 6.25, 95% confidence interval-CI 1.92–10.59, *p* = 0.005), and arterial hypertension (β 5.22, 95% CI 0.22–10.22, *p* = 0.040) were, independently of each other, associated with higher TSS after adjusting for older age, MF phenotype, and cytoreductive treatment.

### 3.3. Impact of Osteoarthritis on Thrombotic Risk

**Figure 2:**
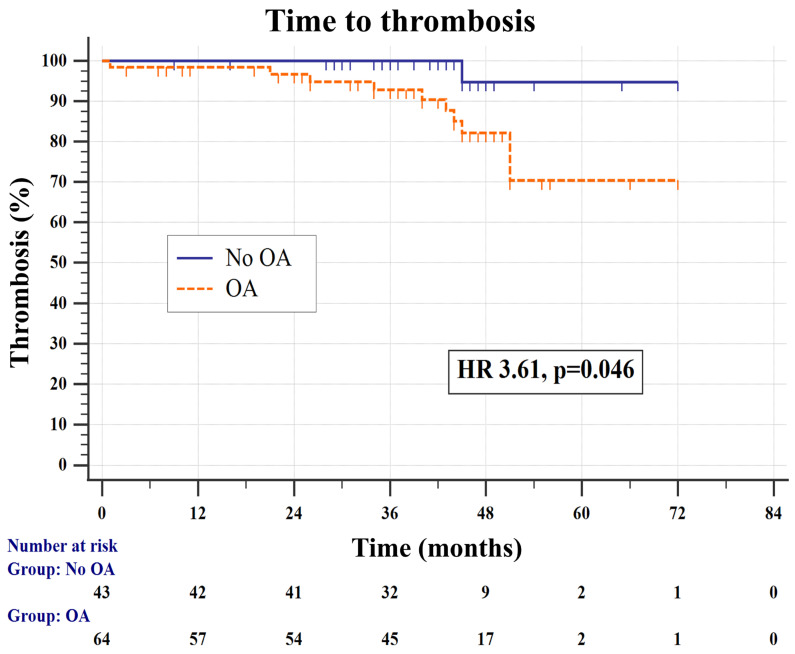
Time to thrombosis stratified according to presence of any osteoarthritis (OA). The Kaplan–Meier method and the log-rank test were used.

Over a median follow-up of 44 months (range 3–72 months), 10 thrombotic events occurred (arterial n = 8, venous n = 2). There were no statistically significant differences in the number of thrombotic events during follow-up (ET = 2/44, PV = 4/35, MF = 4/28; *p* = 0.335) nor in TTT across different MPN disease subtypes (*p* = 0.386). Patients with symptomatic OA of any localization had significantly inferior TTT compared to those without OA (median TTT not reached in both patient groups; hazard ratio-HR 3.61, 95% CI 1.02–12.8, *p* = 0.046), as shown in Figure 2; this observation was evident for hip OA (HR 3.44, *p* = 0.049) and also trended for knee OA (HR 3.36, *p* = 0.056).

Additional univariate predictors of an inferior TTT included age >60 years, prior thrombosis, arterial hypertension, non-aspirin use, and male sex (all *p* values < 0.050). In the multivariable Cox regression model, symptomatic OA of any localization remained independently associated with inferior TTT (HR 15.55, *p* = 0.002), whereas female sex (HR 0.17, *p* = 0.042) and aspirin use (HR 0.15, *p* = 0.019) were protective, after controlling for advanced age, prior thrombosis, arterial hypertension, MF phenotype, and cytoreductive treatment. Both hip (*p* = 0.035) and knee OA (*p* = 0.011) remained independently associated with an inferior TTT in similar multivariable Cox regression models, as shown in Table 2.

To formally evaluate the impact of the limited number of thrombotic events on the stability of the multivariable Cox regression estimates, we performed four additional sensitivity analyses (Table 3). Ridge (L2)-penalized Cox regression, which shrinks coefficient estimates toward the null, markedly attenuated the hazard ratio for any OA from 14.45 to 2.73 (95% CI 0.82–9.09) at a pre-specified penalty of λ = 0.05, and this attenuation became progressively more pronounced with increasing penalty strength (HR ranging from 4.55 to 1.22 across penalty values of 0.02–0.5), confirming that the original point estimate is highly sensitive to model regularization. Elastic-net penalized Cox regression, which combines shrinkage with variable selection, retained OA, female sex, and aspirin use as the only non-zero predictors for the any-OA and hip-OA models (shrunk HR 2.26 and 1.99, respectively), while age, prior thrombosis, arterial hypertension, cytoreductive therapy, and MF subtype were shrunk to zero, indicating limited independent contribution of these covariates given the sparse event data. Bootstrap resampling (1000 iterations, resampling stratified by event status) of the original, non-penalized model yielded a median hazard ratio for any OA of 7.12 (95% percentile CI 1.25–35.96); despite this shrinkage relative to the original point estimate, the bootstrap interval excluded 1 in 98.6% of resamples, supporting a directionally consistent, albeit imprecise, association. Similar patterns were observed for hip and knee OA (Table 3). Finally, a pre-specified parsimonious model restricted to OA, age, sex, and aspirin (events-per-variable ≈ 3.3) yielded hazard ratios broadly consistent with the primary analysis (any OA HR 9.23, 95% CI 1.05–80.91, *p* = 0.045; knee OA HR 8.81, 95% CI 1.46–53.04, *p* = 0.018), although the hip OA estimate no longer reached statistical significance (HR 4.90, 95% CI 0.98–24.57, *p* = 0.054). Taken together, these sensitivity analyses confirm that, while the point estimate of the OA–thrombosis association is unstable and highly sensitive to model specification and penalization—as anticipated given the very low events-per-variable ratio—the direction of the association was consistently replicated across all analytical approaches, further supporting our interpretation of this finding as hypothesis-generating rather than confirmatory.

Model discrimination and calibration were additionally assessed for each multivariable Cox model. Harrell’s concordance index (C-index) was 0.86 for the any-OA model, 0.84 for the hip-OA model, and 0.85 for the knee-OA model, indicating strong apparent discrimination. After bootstrap optimism correction (1000 resamples), the C-index decreased to 0.77, 0.74, and 0.77, respectively, reflecting the expected shrinkage in a dataset with a low events-per-variable ratio, but still indicating acceptable discriminative ability. Bootstrap-derived calibration slopes were 0.77 (any OA), 0.70 (hip OA), and 0.83 (knee OA); values below 1 indicate mild-to-moderate overfitting of the original regression coefficients, consistent with the sensitivity analyses reported above, but do not suggest a fundamentally miscalibrated model.

The proportional hazards assumption was also formally tested for each multivariable Cox model using Schoenfeld residuals. In the any-OA and hip-OA models, no covariate showed significant evidence of a time-varying effect, including OA itself (*p* = 0.223 and *p* = 0.928, respectively; all other covariates *p* ≥ 0.05, with prior thrombosis showing a non-significant trend toward non-proportionality in the any-OA model, *p* = 0.053). In the knee-OA model, the Schoenfeld residuals test suggested a possible violation of the proportional hazards assumption for OA (*p* = 0.047), while all other covariates were compatible with proportionality (*p* > 0.05).

## 4. Discussion

This study demonstrated that the presence of symptomatic OA in MPN patients may be associated with higher symptom burden and, in this preliminary analysis, with elevated thrombotic risk. These findings suggest that OA may not merely co-exist with a more aggressive MPN disease biology, but could also contribute to shaping clinical outcomes.

The association of OA with higher symptom burden is biologically plausible. The inflammatory milieu characterized by elevated circulating IL-1β, IL-6, IL-8, IL-15, TNF-α, and other mediators [7,10] closely overlaps with the cytokine profile driving OA-related synovial inflammation, joint pain, fatigue, and functional impairment [5,6]. The co-occurrence of two chronically inflammatory conditions may therefore produce a synergistic worsening of MPN-SAF items. The independent contribution of female sex and arterial hypertension to higher TSS in our model is consistent with prior data demonstrating greater inflammatory sensitivity and higher cardiovascular comorbidity burden in these subgroups [11,12].

Regarding the observed association of symptomatic OA with higher thrombotic risk, several plausible and inter-related mechanisms may underlie this observation. First, OA-driven systemic inflammation may further amplify the already prothrombotic state inherent to MPNs. Proinflammatory cytokines promote endothelial activation, platelet hyperreactivity, and upregulation of tissue factor—each contributing to a hypercoagulable state [13,14]. In MPN patients, the *JAK2* mutation is associated with a more pronounced inflammatory response and substantially higher thrombotic risk compared to *CALR*-mutated counterparts [15]. The additional inflammatory burden conferred by OA may further shift the hemostatic balance towards thrombosis, particularly in *JAK2*-mutated patients who demonstrated a higher frequency of symptomatic OA [8].

Second, OA-induced physical inactivity and immobility are well-established risk factors for venous thromboembolism in the general population [16]. In MPN patients, who already face an elevated baseline thrombotic risk, OA-related functional limitation may confer an additional and potentially modifiable risk. Furthermore, OA patients in our cohort showed higher use of NSAIDs and opioids. NSAIDs are associated with increased cardiovascular morbidity and may attenuate the antiplatelet effect of aspirin [17], while opioid use can additionally promote stasis and thrombus formation.

Third, OA patients displayed a more adverse cardiometabolic risk profile, with higher rates of arterial hypertension, hyperlipidemia, higher BMI, higher serum uric acid, and lower eGFR—the majority of these comorbidities having been associated with higher thrombotic risk in MPNs in different retrospective datasets [18,19,20,21,22,23,24]. This broader cardiometabolic phenotype may synergize with MPN-related thrombophilia. The independent protective association of aspirin use with TTT further supports this notion, consistent with the well-established role of low-dose aspirin in reducing thrombotic events in ET and PV [1].

Fourth, it is noteworthy that bone disease represents an additional, often underrecognized comorbidity in MPN patients. Emerging evidence demonstrates that MPNs are associated with significant bone morbidity, including increased rates of osteoporosis and osteoporotic fractures [25,26,27,28]. Specifically, patients with MPNs have been shown to carry a markedly elevated risk of fractures compared to the general population [25,26], with distinct MPN subtypes demonstrating differential impact on bone density and fracture risk [26]. The mechanisms underlying bone loss in MPNs likely involve chronic inflammatory cytokine-driven osteoclast activation, JAK-STAT pathway dysregulation, and marrow fibrosis [25]. Given the substantial overlap in risk factors between OA and osteoporosis—including older age, physical inactivity, and chronic inflammation—MPN patients with concomitant OA may face a compounded skeletal vulnerability. These considerations further underscore the importance of comprehensive musculoskeletal assessment in MPN patients.

Finally, several important limitations of this study must be acknowledged. The number of patients and thrombotic events is relatively small, limiting statistical power for both univariate and multivariable survival analyses and resulting in wide confidence intervals, particularly in the Cox regression models. The wide HR observed in the multivariable model likely reflects overfitting due to the low number of events relative to the number of covariates, and these estimates should therefore be interpreted with appropriate caution. With only 10 thrombotic events and eight covariates in the full multivariable Cox model, the events-per-variable (EPV) ratio was approximately 1.2, well below the commonly recommended threshold of 10, which is the most likely explanation for the markedly high and imprecise hazard ratio observed for OA. Given the relatively short follow-up period, limited number of events, and the large number of potential confounders (due to baseline differences in OA vs. non-OA MPN patients), we additionally performed ridge- and elastic-net-penalized Cox regression, bootstrap resampling, and a pre-specified parsimonious reduced model as sensitivity analyses. These analyses confirmed that the point estimate for the OA–thrombosis association is highly sensitive to model specification and the degree of penalization applied, but that the direction of the association remained consistent across all approaches. Nevertheless, formal external validation of this association in larger, adequately powered cohorts, is needed to support our observations.

Other important limitation is the determination of OA status only at baseline (not at follow-up), where some patients who developed OA during follow-up may have been potentially misclassified as OA-negative—this could have affected the exposure status. Also, patients with OA differed from those without OA across several cardiometabolic and inflammatory parameters (age, BMI, hypertension, hyperlipidemia, prior thrombosis, renal function, *JAK2* mutation frequency, and NSAID/opioid use); although these variables were incorporated into multivariable models, residual confounding cannot be excluded, and OA may, at least in part, represent a clinical marker of a broader adverse cardiometabolic and inflammatory phenotype rather than an entirely independent determinant of thrombosis. Therefore, given the small number of events during follow-up, the thrombosis-related findings should be primarily regarded as hypothesis-generating, rather than as evidence that OA per se may be an independent causal or modifiable risk factor for thrombosis. Similarly, several MPN-SAF items—particularly fatigue, inactivity, and bone pain—may directly reflect OA-related joint symptoms rather than MPN disease activity.

Finally, treatment strategies and thrombosis prophylaxis in Croatian community hospitals may differ from those in other healthcare systems, which could affect the generalizability of our findings to other populations. However, a recent national survey has shown that the abidance of Croatian hematologists to international MPN diagnostic and treatment guidelines is very high in both academic and non-academic centers and similar to that of other European countries [29,30,31]. Nevertheless, the two-center Croatian cohort may not fully represent the broader MPN population, and findings should be confirmed in larger, multicenter studies also including other demographic populations.

Lastly, longitudinal MPN-SAF assessments were not performed, precluding an evaluation of symptom trajectory over time. Inflammatory cytokine profiling was also not included—this would have helped to unravel the potential pathophysiological mechanisms underlying the observed associations. However, the consistency of findings across both hip and knee OA localizations and across univariate and multivariable models may strengthen the validity of the presented observations.

## 5. Conclusions

Symptomatic OA may be associated with higher symptom burden and, in this exploratory analysis, with higher thrombotic risk in patients with MPNs. Given the small number of thrombotic events and the large number of confounders, it is difficult to interpret this association as a direct proof of an independent causal effect of OA on thrombosis and the results from this study should thus be externally validated. Nevertheless, despite these limitations, the presented findings argue for systematic OA evaluation as a component of routine MPN clinical care and underscore the importance of multidisciplinary management with proactive OA recognition and targeted interventions, including weight reduction, physical rehabilitation, and optimization of cardiovascular risk factors. Further prospective studies in larger patient cohorts with comprehensive cytokine profiling and longitudinal assessments are needed to confirm these results and to elucidate the exact underlying pathophysiological mechanisms.

## Figures and Tables

**Table 1:** Patient characteristics stratified according to the presence of symptomatic osteoarthritis (OA).

Variable	Total (*n* = 107)	Any OA (*n* = 64, 59.8%)	No OA (*n* = 43, 40.2%)	*p* *
Age, years (median, range)	68 (35–90)	70 (41–90)	61 (35–79)	**<0.001**
Female sex	58 (54.2%)	37 (57.8%)	21 (48.8%)	0.363
Disease duration, years (median, range)	2 (0–20)	2 (0–20)	2 (0–15)	0.827
Newly diagnosed	29 (27.1%)	18 (28.1%)	11 (25.6%)	0.772
ET	44 (41.1%)	22 (50%)	22 (50%)	0.176 0.064 *
PV	35 (32.7%)	22 (62.9%)	13 (37.1%)	
MF	28 (26.2%)	20 (71.4%)	8 (28.6%)	
*JAK2-V617F*	78 (72.9%)	52 (66.7%)	26 (33.3%)	0.053 0.043 *
*CALR*	16 (15%)	6 (37.5%)	10 (62.5%)	
Negative	13 (12.1%)	6 (46.2%)	7 (53.8%)	
*JAK2* vs. other mutation/negative	78 (72.9%)	52 (81.2%)	26 (60.5%)	**0.018**
Bone marrow fibrosis grade 1–3	47 (43.9%)	31 (48.4%)	16 (37.2%)	0.255
Total symptoms score (median, range)	16 (0–50)	20 (0–50)	11 (0–43)	**<0.001**
ECOG 2–4	22 (20.6%)	19 (29.7%)	3 (7%)	**0.004**
Previous thrombosis	22 (20.6%)	18 (28.1%)	4 (9.3%)	**0.018**
Body weight, kg, mean ± SD	77.4 (±14.1)	81.4 (±13.4)	70.6 (±12.5)	**0.001**
Body height, m, mean ± SD	1.69 (±8.8)	169.4 (±9.1)	168.9 (±8.4)	0.818
BMI, kg/m^2^, mean ± SD	26.9 (±4.2)	28.3 (±3.9)	24.6 (±3.6)	**<0.001**
Arterial hypertension	66 (61.7%)	46 (71.9%)	20 (46.5%)	**0.008**
Diabetes mellitus	16 (15%)	12 (18.8%)	4 (9.3%)	0.181
Hyperlipidemia	41 (38.3%)	31 (48.4%)	10 (23.3%)	**0.008**
Smoking	22 (20.6%)	9 (14.1%)	13 (30.2%)	**0.043**
Autoimmune diseases	3 (2.8%)	2 (3.1%)	1 (2.3%)	1
Gout	6 (5.6%)	5 (7.8%)	1 (2.3%)	0.398
Cytoreduction	81 (75.7%)	49 (76.6%)	32 (74.4%)	0.800
Hydroxyurea	73 (68.2%)	47 (73.4%)	26 (60.5%)	0.159
Aspirin	82 (76.6%)	48 (75%)	34 (79.1%)	0.627
Oral anticoagulants	12 (11.2%)	10 (15.6%)	2 (4.7%)	0.177
NSAIDs	37 (34.6%)	28 (43.7%)	9 (20.9%)	**0.015**
Opioids	18 (16.8%)	15 (23.4%)	3 (7%)	**0.026**
Corticosteroids	4 (3.8%)	4 (6.2%)	0%	0.150
Statins	34 (31.8%)	27 (42.2%)	7 (16.3%)	**0.005**
ACE inhibitors	52 (48.6%)	38 (59.4%)	14 (32.6%)	**0.006**
Leukocytes, ×10^9^/L (median, range)	7.6 (2.9–24)	7.6 (2.9–24)	8.1 (3.8–14.5)	0.615
Granulocytes, ×10^9^/L (median, range)	5.2 (1.5–17.4)	5.1 (1.5–17.4)	5.3 (1.8–13.2)	0.694
Lymphocytes, ×10^9^/L (median, range)	1.6 (0.7–4.4)	1.45 (0.7–4.4)	1.6 (0.7–4)	0.598
Monocytes, ×10^9^/L (median, range)	0.5 (0.1–3.9)	0.5 (0.1–1.6)	0.5 (0.2–3.9)	0.796
Eosinophils, ×10^9^/L (median, range)	0.1 (0–4.1)	0.1 (0–4.1)	0.1 (0.1–0.9)	0.638
Basophils, ×10^9^/L (median, range)	0.1 (0–0.8)	0.1 (0–0.6)	0.1 (0–0.8)	0.126
Hemoglobin, g/L (median, range)	139 (79–192)	136 (79–192)	142 (86–162)	**0.004**
Hematocrit, % (median, range)	43 (25–61.8)	42.5 (25–61.8)	44 (27–59)	**0.029**
Platelets, ×10^9^/L (median, range)	407 (56–1293)	401.5 (56–1293)	469 (133–794)	0.229
LDH, IU/L (median, range)	226 (123–913)	231 (123–913)	212 (173–823)	0.171
C-reactive protein, mg/L (median, range)	1.7 (0–28.5)	1.4 (0–28.5)	1.9 (0.3–6.2)	0.886
Uric acid, μmol/L (median, range)	308 (89–680)	321 (177–680)	252 (89–517)	**0.001**
eGFR, mL/min/1.73 m^2^ (median, range)	74.8 (30.1–1508)	72 (30.1–124.8)	81 (41.5–1508)	**0.007**

Statistically significant *p* values are bolded and set at <0.050; * chi-square trend test. OA = osteoarthritis, ET = essential thrombocythemia, PV = polycythemia vera, MF = myelofibrosis, *JAK2* = Janus Kinase 2, *CALR* = calreticulin, SD = standard deviation, ECOG = Eastern Cooperative Oncology Group, NSAIDs = non-steroidal anti-inflammatory drugs, ACE-i = angiotensin-converting enzyme inhibitors, LDH = serum lactate dehydrogenase, eGFR = estimated glomerular filtration rate.

**Table 2:** Multivariate Cox regression analysis of variables associated with time to thrombosis for any osteoarthritis (OA), hip OA, and knee OA.

Any OA				Hip OA				Knee OA			
*Variable*	*HR*	*95% CI*	*p*	*Variable*	*HR*	*95% CI*	*p*	*Variable*	*HR*	*95% CI*	*p*
Any OA	15.55	1.46–14.96	**0.022**	Hip OA	6.91	1.24–38.5	**0.027**	Knee OA	22.2	2.82–172	**0.003**
Age	0.92	0.85–1.03	0.186	Age	0.94	0.87–1.02	0.150	Age	0.89	0.81–0.97	**0.013**
Female sex	0.17	0.03–0.94	**0.042**	Female sex	0.18	0.03–0.92	**0.040**	Female sex	0.18	0.03–0.095	**0.044**
Aspirin	0.15	0.03–0.74	**0.019**	Aspirin	0.21	0.04–0.94	**0.042**	Aspirin	0.12	0.02–0.757	**0.007**
Prior thrombosis	0.59	0.15–3.04	0.618	Prior thrombosis	0.72	0.15–3.44	0.685	Prior thrombosis	1.31	0.28–6.12	0.727
Arterial hypertension	4.15	0.56–16.01	0.197	Arterial hypertension	3.37	0.61–18.68	0.163	Arterial hypertension	2.22	0.41–12	0.545
Cytoreduction	2.87	0.24–21.86	0.459	Cytoreduction	2.37	0.22–24.63	0.468	Cytoreduction	3.53	0.34–34.1	0.275
MF subtype	1.20	0.45–2.75	0.802	MF subtype	1.02	0.23–4.49	0.969	MF subtype	0.30	0.52–3.15	0.580

Statistically significant *p* values are bolded and set to <0.050. HR = hazard ratio; CI = confidence interval; OA = osteoarthritis; MF = myelofibrosis.

**Table 3:** Sensitivity and penalized regression analyses of the association between osteoarthritis (OA) and time to thrombosis.

Analytical Approach	Any OA HR (95% CI)	Hip OA HR (95% CI)	Knee OA HR (95% CI)
Original multivariable Cox model (7 covariates; Table 2)	14.45 (1.45–144.04)	6.30 (1.12–35.56)	10.94 (1.65–72.54)
Ridge (L2)-penalized Cox regression (λ = 0.05)	2.73 (0.82–9.09)	2.39 (0.79–7.23)	2.75 (0.89–8.53)
Elastic-net penalized Cox regression (cross-validated) †	2.26	1.99	7.20
Bootstrap resampling (1000 iterations) Median HR (95% percentile CI)	7.12 (1.25–35.96)	4.83 (1.13–27.43)	7.91 (1.42–46.11)
Parsimonious model (OA, age, sex, aspirin; EPV ≈ 3.3)	9.23 (1.05–80.91)	4.90 (0.98–24.57)	8.81 (1.46–53.04)

HR = hazard ratio; CI = confidence interval; OA = osteoarthritis; EPV = events-per-variable. † Elastic-net estimates are shrunk point coefficients derived from cross-validated coordinate-descent regularization; analytic confidence intervals are not defined for penalized partial-likelihood estimates.

## Data Availability

The data that support the findings of this study are available from the corresponding authors upon reasonable request (the data are not publicly available due to ethical restrictions.).
